# Single-cell analysis of CD14^+^CD16^+^ monocytes identifies a subpopulation with an enhanced migratory and inflammatory phenotype

**DOI:** 10.3389/fimmu.2025.1475480

**Published:** 2025-02-20

**Authors:** Vanessa Y. Ruiz, Tina M. Calderon, Rosiris Leon-Rivera, Vanessa Chilunda, Jinghang Zhang, Joan W. Berman

**Affiliations:** ^1^ Department of Pathology, Albert Einstein College of Medicine, New York, NY, United States; ^2^ Department of Microbiology and Immunology, Albert Einstein College of Medicine, New York, NY, United States

**Keywords:** CD14^+^CD16^+^ monocytes, intermediate monocytes, BBB, scRNA-seq, ROS, cytokines

## Abstract

Monocytes in the central nervous system (CNS) play a pivotal role in surveillance and homeostasis, and can exacerbate pathogenic processes during injury, infection, or inflammation. CD14^+^CD16^+^ monocytes exhibit diverse functions and contribute to neuroinflammatory diseases, including HIV-associated neurocognitive impairment (HIV-NCI). Analysis of human CD14^+^CD16^+^ monocytes matured *in vitro* by single-cell RNA sequencing identified a heterogenous population of nine clusters. Ingenuity pathway analysis of differentially expressed genes in each cluster identified increased migratory and inflammatory pathways for a group of clusters, which we termed Group 1 monocytes. Group 1 monocytes, distinguished by increased ALCAM, CD52, CD63, and SDC2, exhibited gene expression signatures implicated in CNS inflammatory diseases, produced higher levels of CXCL12, IL-1Ra, IL-6, IL-10, TNFα, and ROS, and preferentially transmigrated across a human *in vitro* blood-brain barrier model. Thus, Group 1 cells within the CD14^+^CD16^+^ monocyte subset are likely to be major contributors to neuroinflammatory diseases.

## Introduction

The central nervous system (CNS) is a dynamic immune landscape composed of resident and peripheral immune cells, which can exert both protective and detrimental effects on this vulnerable environment (1). Monocytes are peripheral bone marrow derived myeloid cells composed of heterogeneous subsets that surveil the CNS in response to infection or damage, actively crossing the blood-brain barrier (BBB) (2, 3). In the bloodstream, some monocytes undergo maturation, transitioning from classical (CD14^+^), to intermediate (CD14^+^CD16^+^), and finally to non-classical (CD14^lo^CD16^+^) subsets (4). Despite their low levels in the CNS under steady-state conditions (3), monocytes have been implicated in various neuroinflammatory diseases including HIV-associated neurocognitive impairment (HIV-NCI), Alzheimer’s disease (AD), multiple sclerosis (MS), and Parkinson’s disease (5–7), although their precise contributions to the neuropathogenesis of these diseases remain incompletely understood.

CD14^+^CD16^+^ monocytes represent a mature subset that comprises 5-10% of circulating monocytes. These monocytes have been characterized as more inflammatory than the other two subsets, and increase rapidly in number in response to injury, infection, or inflammation (8–11). CD14^+^CD16^+^ monocytes specifically have been implicated in HIV-NCI (12–14), where they can increase and comprise up to 50% of total monocytes in the blood of people with HIV (PWH) (9, 12, 15). They also preferentially transmigrate across the BBB compared to other monocyte subsets (16–19). Additionally, increased circulating levels of CD14^+^CD16^+^ monocytes have been associated with cardiovascular diseases including atherosclerosis, heart failure, myocardial infarction, and coronary artery disease (CAD) (20–25). Despite widespread recognition of the three major monocyte subsets, there is conflicting evidence regarding the functions of CD14^+^CD16^+^ monocytes and their roles in pathological processes. Several studies demonstrated additional monocyte subsets beyond the traditional three, with some studies citing from five and even nine subsets of peripheral blood monocytes (26–29). Some of these studies may not be identifying additional subsets, but rather unique subpopulations within the three major monocyte subsets, particularly CD14^+^CD16^+^ monocytes. In addition, the conflicting results reported by studies characterizing functional properties of CD14^+^CD16^+^ monocytes led us to hypothesize that this subset is heterogenous, with subpopulations of cells exhibiting varying functional characteristics.

To gain a more complete understanding of the contributions of CD14^+^CD16^+^ monocytes to CNS inflammatory processes, and specifically HIV-NCI, we characterized in detail this heterogeneous group of monocytes. Peripheral human CD14^+^ monocytes were matured *in vitro* to CD14^+^CD16^+^ monocytes and analyzed by single-cell RNA sequencing (scRNA-seq), identifying nine distinct cell clusters. Ingenuity pathway analysis (IPA) of distinct differentially expressed genes (DEGs) in each cluster compared to all other clusters, identified two groups with differing functional characteristics. Clusters 2, 4, and 8 exhibited increased migratory and inflammatory pathways compared to all other clusters. We termed monocytes from clusters 2, 4, and 8 as Group 1 monocytes, and cells from the remaining clusters as Group 2 monocytes. Genes involved in migratory and inflammatory pathways that were identified by scRNA-seq for their increased expression in Group 1 compared to Group 2 monocytes were validated by flow cytometry studies. Group 1, unlike Group 2, monocytes were characterized by surface expression of the junctional adhesion protein ALCAM, in combination with increased levels of CD52, CD63, and SDC2. Characterization of functional properties of these cells showed that Group 1 monocytes produced more CXCL12, IL-1Ra, IL-6, IL-10, TNFα, and reactive oxygen species (ROS) compared to Group 2 monocytes. Additionally, Group 1 monocytes preferentially transmigrated across an *in vitro* model of the human BBB to CCL2 compared to Group 2 cells.

This comprehensive characterization demonstrates significant phenotypic and functional heterogeneity within the CD14^+^CD16^+^ monocyte subset. These data suggest that Group 1 monocytes have an increased capacity to transmigrate across the BBB into the CNS and mediate neuroinflammation, contributing to neuronal damage and loss. These findings form the foundation for development of targeted therapeutic interventions to reduce, or even eliminate, the neurotoxic impact of this specific subpopulation of CD14^+^CD16^+^ monocytes.

## Materials and methods

### Isolation and enrichment of CD14^+^CD16^+^ monocytes

Human peripheral blood mononuclear cells (PBMCs) were obtained from leukopaks of healthy donors (New York Blood Center and Texas Gulf Coast Regional Blood Center) using Ficoll-Paque PLUS (Fisher Scientific) density gradient centrifugation. CD14^+^ monocytes were magnetically labeled using CD14 MicroBeads (Miltenyi) and positively selected with magnetic nanoparticle cell separation (STEMCELL). CD14^+^ monocytes were resuspended at 2×10^6^ cells/ml in monocyte media consisting of RPMI 1640 supplemented with 10% human AB serum (Sigma), 5% fetal bovine serum (FBS) (Gibco, Thermo Fisher), 1% HEPES (Teknova), 1% Pen-Strep (Gibco, Thermo Fisher), and 10 ng/ml macrophage colony-stimulating factor (M-CSF) (Peprotech). Cells were cultured non-adherently for 3 days at 37°C with 5% CO_2_ in Teflon-coated flasks (Thermo Fisher) to increase the population of CD14^+^CD16^+^ monocytes, as previously described (16, 17, 19, 30). In our previously published study (31), uninfected monocytes from two donors were matured *in vitro* and sorted for CD14^+^CD16^+^ intermediate monocytes, followed by scRNA-seq analysis. While this analysis was performed on cell populations highly enriched for intermediate monocytes, there may have been a very small percentage of non-classical monocytes after sorting. In the current study, this previously published scRNA-seq data set was re-analyzed and used for DEG analysis to identify Group 1 and 2 monocytes. Monocytes from new sets of donors were matured *in vitro* to obtain CD14^+^CD16^+^ monocytes for subsequent experiments to characterize Group 1 and 2 monocytes.

### scRNA-seq monocyte clustering

We re-analyzed scRNA-seq data from CD14^+^CD16^+^ monocytes of two donors matured in culture from our previously published study (31) using Seurat (v3.2.2) package on R programming language (v4.1.0). Cells were confirmed for CD14 and CD16 positivity by flow cytometry before and after scRNA-seq analysis (31). Quality control was performed on each individual sample to remove cells with high mitochondrial gene expression and cells with less than 200 expressed genes (31). Samples from both donors were merged to yield a total of 26,977 cells for downstream analyses. Merged data was normalized using the SCtransform function (31, 32). The merged data were then normalized and integrated, as previously described (31, 32). To identify cell clusters, we first performed dimensionality reduction by principal component analysis (PCA) using the top 19 principal components (PCs). Clusters were generated from PCs using the FindClusters function with resolution set at 0.3. The clusters were then visualized using the uniform manifold approximation and projection (UMAP) dimensionality reduction algorithm.

### Differential gene expression and molecular pathway analysis

DEGs for each cluster of CD14^+^CD16^+^ monocytes were identified by the FindMarkers function in the Seurat package, as previously described (31, 32). For this study, expression of genes in each individual cluster were compared to the expression of the same genes in all the remaining clusters, to quantify an average difference (avg diff) in expression for each DEG. We performed DEG analysis on Pearson’s residuals after regularized negative binomial regression, employing t-tests per gene for all genes identified in at least 3 cells in the groups being compared. Genes were considered differentially expressed if they had an adjusted p-value<0.005 and FDR<0.01 (31, 32) (**Supplementary Table S1**). Significant DEGs were then analyzed using IPA software (Qiagen). We used a cut off Z-score of log_2_fold-change ± 0.3 and FDR<0.05 to correlate significant avg diff for DEGs in each cluster with pathways and functions involved in motility, chemotaxis, migration, invasiveness, and inflammation as determined by Z-score.

### Surface marker staining and analysis by flow cytometry

For analysis of monocyte surface markers, CD14^+^CD16^+^ monocytes from ten new independent donors were cultured as described above, washed once with 1X PBS, and stained with Zombie NIR fixable viability dye (Biolegend, 1:1000) for 30 minutes at room temperature (RT) in the dark. Cells (0.2-1×10^6^ cells/tube) were washed in FACS staining buffer consisting of 1X PBS with 1% bovine serum albumin (BSA), and stained with surface antibodies Alexa-700 anti-human CD14 (BD Biosciences, clone M5E2, 1.2μg), PE-Cy7 anti-human CD16 (BD Biosciences, clone 3GB, 3.2μg), BB515 anti-human ALCAM/CD166 (BD Biosciences, clone 3A6, 1.8μg), PE anti-human CD52 (Biolegend, clone HI186, 0.45μg), PerCP-Cy5.5 anti-CD63 (BD Biosciences, clone H5C6, 1.2μg), and APC anti-human SDC2/CD362 (R&D systems, clone 305515, 0.07μg) for 30 minutes at 4°C in the dark. These antibodies were used in all experiments involving staining of CD14^+^CD16^+^ monocytes, unless stated otherwise. Cells were then washed with FACS staining buffer and fixed with 2% paraformaldehyde (PFA) in 1X PBS supplemented with 5mM EDTA. Fluorescence minus one (FMO) controls were prepared concomitantly. The AbC Total Antibody Compensation Bead Kit (Invitrogen) was used to prepare bead compensation controls. Samples were acquired on the Attune NxT flow cytometer (Thermo Fisher). Data was analyzed using FlowJo v10.10.0 software (BD Biosciences) with only CD14^+^CD16^+^ monocytes gated in Quadrant 2 used for subsequent analyses. Additionally, cells excluded from singlets and live/dead gates were backgated and determined to be dying cells and/or debris as determined by their FSC/SSC positions and were not included in subsequent analyses.

### Fluorescence-activated cell sorting

Single cell suspensions of matured CD14^+^CD16^+^ monocytes from 13 new independent donors were stained in polypropylene FACS tubes that were previously coated with PBS with 4% FBS (Gibco, Thermo Fisher) overnight at 4°C. Flow cytometry staining was performed for CD14, CD16, ALCAM, and viability, as described above. Monocytes to be sorted were seeded at 5-10×10^6^ cells/tube, stained, and then pooled and re-suspended at 15×10^6^ cells/ml in FACS sorting buffer consisting of 1X PBS supplemented with 2mM EDTA, 25mM HEPES (Teknova), 4% FBS (Gibco, Thermo Fisher), and 1% Pen-Strep (Gibco, Thermo Fisher). Samples were sorted on the BD FACS Aria in the Albert Einstein College of Medicine (AECOM) Flow Cytometry Core. Gates were set using FMO controls to sort CD14^+^CD16^+^ALCAM^+^ and CD14^+^CD16^+^ALCAM^-/lo^ populations. Cells were sorted by ALCAM expression without the use of other surface proteins that define Group 1 to obtain sufficient number of cells for subsequent RNA extraction and qRT-PCR analyses. Sorted cells were collected in BD Falcon tubes containing monocyte media supplemented with M-CSF and kept at 4°C until RNA isolation, which was performed the same day.

### RNA isolation and qRT-PCR

To compare CD14^+^CD16^+^ALCAM^+^ and CD14^+^CD16^+^ALCAM^-/lo^ populations, RNA was extracted from sorted populations using TRIzol (Invitrogen), per the manufacturer’s protocol. The number of cells isolated after sorting CD14^+^CD16^+^ALCAM^+^ and CD14^+^CD16^+^ALCAM^-/lo^ monocyte populations from different donors varied greatly, with monocytes from some donors yielding large and others yielding small numbers of sorted cells. As a result, not enough RNA was isolated to quantify expression of all the markers by qRT-PCR for cells from every donor. RNA concentration and purity was evaluated using a NanoDrop 2000 spectrophotometer (Thermo Fisher). Only samples with 260/280 values at ~2 were used. RNA quality was assessed with the Agilent 2100 Bioanalyzer (AECOM Genomics Core), with RNA quality numbers all above 8. One to 2μg of RNA was converted to cDNA using the SuperScript VILO Master Mix (Invitrogen), per the manufacturer’s protocol. QRT-PCR was performed using the QuantStudio 3 real-time PCR system and software (Applied Biosystems). Relative expression of target genes between CD14^+^CD16^+^ALCAM^+^ and CD14^+^CD16^+^ALCAM^-/lo^ cells was quantified using TaqMan^®^ probes (Applied Biosystems) and normalized to internal control gene *18S* using the 2^-ΔΔCt^ method. Each comparison was performed in triplicate using 1μl of cDNA. A fold change of expression between CD14^+^CD16^+^ALCAM^+^ and CD14^+^CD16^+^ALCAM^-/lo^ populations was generated by setting CD14^+^CD16^+^ALCAM^-/lo^ populations to one. The following TaqMan^®^ primer probes from Thermo Fisher were used (catalog: 4331182): *18S* (Hs99999901_s1), *ALCAM* (Hs00233455_m1), *APOC1* (Hs00155790_m1), *APOE* (Hs00171168_m1), *CHI3L1* (Hs01072228_m1), *CLEC7A* (Hs01902549_s1), *CTSB* (Hs00947433_m1), *DUSP1* (Hs00610256_g1), *FCGR2B* (Hs01634996_s1), *LGALS3* (Hs00173587_m1), *SPP1* (Hs00959010_m1), and *TPT1* (Hs02621289_g1). PCR primer efficiencies were evaluated using 5-fold serial dilutions of cDNA isolated from mature monocytes to generate standard curves, with all target efficiencies between 90-110%.

### Intracellular mediator detection by flow cytometry

For intracellular staining, CD14^+^CD16^+^ monocytes from ten new independent donors were matured as described previously, with 100 ng/ml LPS (Sigma-Aldrich) added for the last 24 hours, and 10 μg/ml Brefeldin A (eBioscience) added for the last 4 hours, of the three-day incubation. Cells (1×10^6^ cells/sample) were then added to a 96-well U bottom plate, washed, stained for viability with Zombie NIR (1:4000), and then stained for surface markers CD14, CD16, and Alexa-488 anti-human ALCAM/CD166 (Bio-Rad, clone 3A6, 0.4μg), as described above. Cells were fixed and permeabilized using the Foxp3 Fixation/Permeabilization working solution from the Foxp3/Transcription Factor Staining Buffer Set (eBioscience) for 1 hour in the dark on ice. Cells were then washed with 1X Permeabilization buffer (eBioscience), and stained intracellularly for one hour in the dark on ice with the following antibodies diluted in 1X Permeabilization buffer and Brilliant Stain Buffer (BD Biosciences, 1:20): PE mouse anti-human IL-1R antagonist (BD Biosciences, clone AS17, 0.05μg), PerCP-Cy5.5 mouse anti-human IL-6 (Biolegend, clone MQ2-13A5, 0.125μg), PE-CF594 mouse anti-human IL-8 (R&D, clone 79018, 0.013μg), BV711 mouse anti-human IL-10 (BD Biosciences, clone JES3-9D7, 0.325μg), RB780 mouse anti-human TNFα (BD Biosciences, clone Mab11, 0.325μg). Cells were acquired using spectral cytometry on a Cytek Aurora in the AECOM Flow Cytometry Core. For these experiments, the percent expression of intracellular mediators was compared between CD14^+^CD16^+^ALCAM^+^ and CD14^+^CD16^+^ALCAM^-/lo^ cells, without the use of other surface proteins that define Group 1, because of the extensive antibody panel used to assess all the mediators in each cell per sample, and the number of cells needed for necessary controls.

### ROS assay by flow cytometry

To measure ROS, the CellROX Deep Red Flow Cytometry Assay kit (Invitrogen) was used according to the manufacturer’s instructions. Briefly, matured CD14^+^CD16^+^ monocytes from 11 new independent donors were added to FACS tubes (0.4-1×10^6^ cells/sample). Positive controls were treated with 200μM tert-butyl hydrogen peroxide (TBHP) and negative controls with 1mM N-Acetylcysteine (NAC) for 1 hour in the dark at 37°C, prior to measurement of ROS. After incubation, cells were washed with 1X PBS and subsequently incubated with 5μM CellROX Deep Red reagent for 1 hour at 37°C in the dark. Cells were then washed and stained with Zombie NIR fixable viability stain (Biolegend) for dead cell exclusion, followed by staining with antibodies to cell surface ALCAM, CD14, CD16, CD52, and CD63, as described above. ROS was identified by the corresponding median fluorescence intensity (MFI) detected at emission/excitation wavelength 644/665 nm by the red laser. Samples were acquired on the Attune NxT flow cytometer (Thermo Fisher). Baseline ROS MFI was compared between CD14^+^CD16^+^ALCAM^+^CD52^hi^CD63^hi^ and CD14^+^CD16^+^ALCAM^-/lo^CD52^lo^CD63^lo^ populations. Designations of high and low expression of CD52 and CD63 were defined by the top and bottom 20^th^ percentiles in that fluorophore.

### BBB transmigration experiments

We used a human *in vitro* BBB model to study transmigration of CD14^+^CD16^+^ monocytes. This model is a co-culture system of human BMVEC (Cell Systems) and human cortical astrocytes separated by a gelatin-coated tissue culture insert with 3μm pores (BD Falcon) that are placed in the wells of a 24-well plate containing media (baseline transmigration) or media with CCL2 (200ng/ml; R&D systems), as described previously (16, 30, 33). BMVEC seeded on the upper surface of the tissue culture represent the peripheral side of the BBB. Primary human astrocytes seeded on the underside represent the CNS side. A portion of matured CD14^+^CD16^+^ monocytes from six new independent donors (pre-transmigrated cells) was stained with antibodies to CD14, CD16, ALCAM, CD52, CD63, SDC2, with FMO controls, as described above. Three × 10^5^ cells were added to each BBB co-culture and allowed to transmigrate to CCL2 for 24 hours, with each condition performed in quadruplicate. Permeability of co-cultures was assessed using Evans blue dye coupled to BSA (16, 19, 33) and all co-cultures were impermeable. Transmigrated cells were collected and stained with the same panel of antibodies as the pre-transmigration cells. At least 20,000 events were collected for pre-transmigrated cells, and the entire sample of post-transmigrated cells was collected for analysis by flow cytometry. Positivity for each marker was determined by FMO controls on the pre-transmigration population, with the same gating applied to the post-transmigrated cells. The number of cells that transmigrated from each Group was expressed as the percent of transmigrated cells relative to the input population of each Group of monocytes, as described previously (19, 34). Group 1 monocyte surface proteins were evaluated individually and sequentially on CD14^+^CD16^+^ALCAM^+^ and CD14^+^CD16^+^ALCAM^-/lo^ populations. CD14^+^CD16^+^ monocytes were first gated for ALCAM^+^ and ALCAM^-/lo^ cells, and then high and low expression of each additional surface protein marker was assessed separately on these two populations. High and low expression was defined as the top and bottom 20^th^ percentiles in that fluorophore. Additionally, the proportion of Group 1 monocytes that were CD14^+^CD16^+^ALCAM^+^CD52^+^CD63^+^SDC2^+^ was compared pre- and post-transmigration using sequential gating. Overall positivity, and not high expression, of each marker was used to identify Group 1 monocytes because of the limited number of transmigrated cells available for analysis of high expression of all six markers in each cell by sequential gating.

### Statistical analysis

Statistical analyses and graphs were generated with GraphPad Prism software v.10.1.0 (GraphPad software). Normality was assessed using D’Agostino & Pearson’s tests. For normally distributed data, paired student t-tests and one-sample t-tests were used to detect significant differences between groups. For non-normally distributed data, Wilcoxon matched-pairs signed rank and Wilcoxon signed rank test were used to detect significant differences between groups. Data was presented as means ± standard error of the mean (SEM).

## Results

### Single-cell RNA sequencing of CD14^+^CD16^+^ monocytes matured *in vitro* identifies nine distinct clusters with one group of clusters exhibiting an increased migratory and inflammatory phenotype

**Figure 1:**
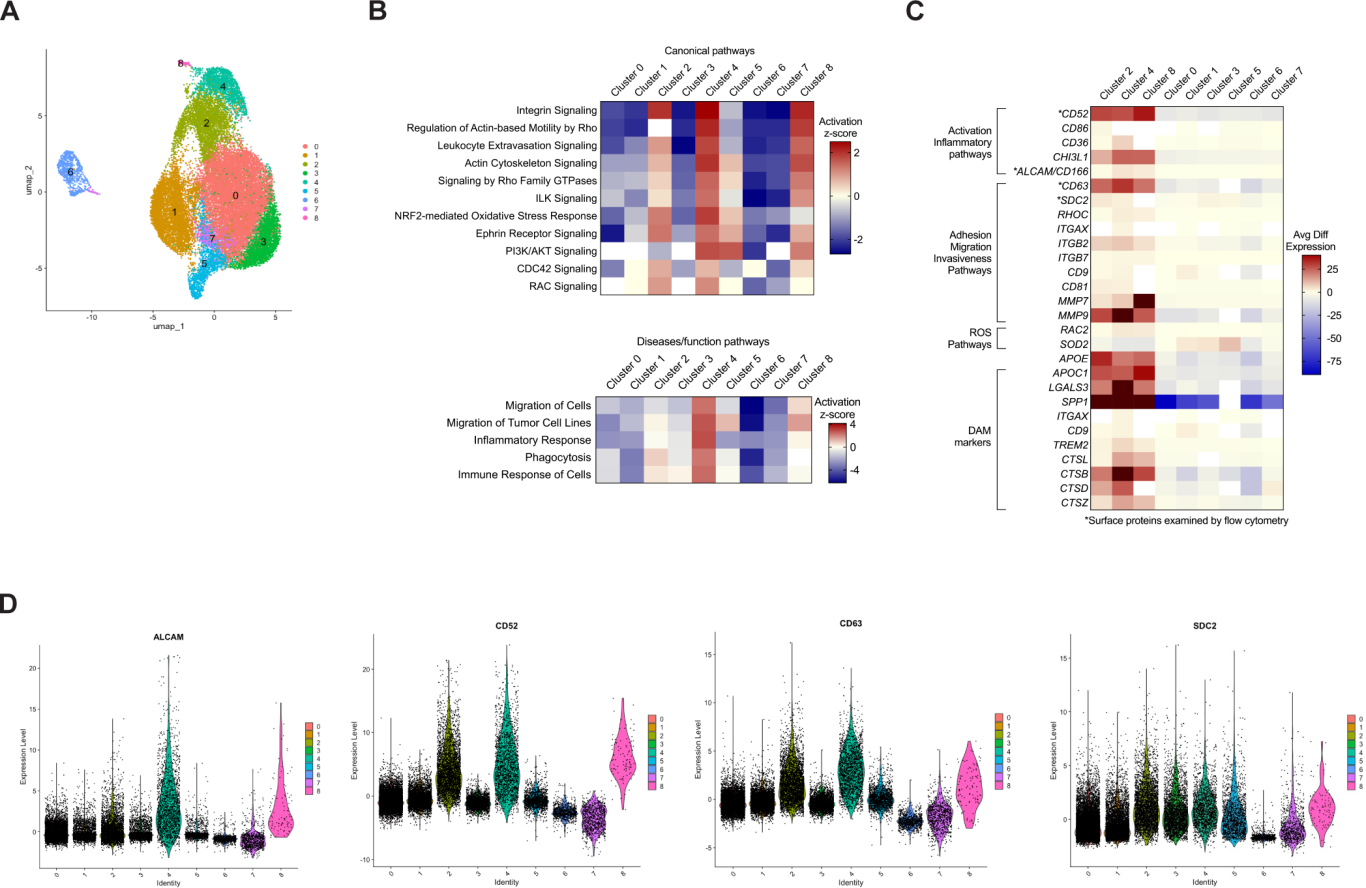
Analysis of CD14^+^CD16^+^ monocytes by scRNA-seq identifies nine clusters with one group of clusters exhibiting an increased migratory and inflammatory phenotype. Human CD14^+^ monocytes were isolated from leukopaks and cultured non-adherently with M-CSF *in vitro* for three days to obtain mature CD14^+^CD16^+^ monocytes, followed by cryopreservation for subsequent scRNA-seq analysis. **(A)** UMAP plot of 26,977 cells merged from two independent donors show nine clusters of monocytes, with each color representing a different cluster designated by number. The number of cells in each cluster are found in **Supplementary Table S2**. **(B)** Heatmap of IPA of DEGs in each cluster compared to all other clusters, showing the level of expression of canonical and disease/functions pathways in each cluster as determined by Z-scores. Red or blue is indicative of increased and decreased pathway expression, respectively, with white denoting insufficient significant DEGs to analyze with IPA. **(C)** Heatmap of selected DEGs involved in migratory or inflammatory pathways, or markers of DAM, showing avg diff in expression of genes in each cluster compared to all other clusters. The color scale is based on distribution of avg diff values, with red representing positive changes, dark blue representing negative changes, and white representing insignificant changes in avg diff. **(D)** Violin plots showing relative expression of DEGs that encode surface proteins ALCAM, CD52, CD63, and SDC2. Each dot represents an individual cell, and each color corresponds to a cluster. The full list of DEGs in each cluster can be found in **Supplementary Table S1**. UMAP, uniform manifold approximation and projection, IPA, ingenuity pathway analysis, DEGs, differentially expressed genes, DAM, disease-associated microglia, avg diff, average difference in expression.

CD14^+^CD16^+^ monocytes are implicated in various pathological processes and diseases (10, 11, 24). To characterize different subpopulations within this heterogenous monocyte subset, we performed new analysis of scRNA-seq data from a previous study that focused on characterizing gene expression in CD14^+^ monocytes matured *in vitro* to CD14^+^CD16^+^ monocytes, and subsequently infected with HIV (31). In this previous study, uninfected CD14^+^CD16^+^ monocytes from two independent donors were analyzed by scRNA-seq as a control. In the current study, we reanalyzed this scRNA-seq data to examine subpopulations within uninfected CD14^+^CD16^+^ monocytes. We assessed an average of 13,489 cells per donor, with a total of 26,977 cells (**Supplementary Table S2**). Clustering analysis and UMAP embedding showed that CD14^+^CD16^+^ monocytes segregated into nine clusters (**Figure 1A**), indicating heterogeneity within the intermediate monocyte subset. The data was then analyzed to quantify expression of genes in each individual cluster compared to that of all the remaining clusters to identify DEGs. IPA was then performed to indicate molecular pathways that were enriched in each cluster compared to all other clusters. IPA identified increased migratory and inflammatory pathways, including integrin signaling, migration of cells and of tumor cell lines, leukocyte extravasation signaling, actin cytoskeleton signaling, and inflammatory response, in clusters 2, 4, and 8 (**Figure 1B**). We termed cells in clusters 2, 4, and 8 as Group 1 monocytes and cells in clusters 0, 1, 3, 5, 6, and 7 as Group 2 monocytes. Of the CD14^+^CD16^+^ monocytes analyzed by scRNA-seq, approximately 19% were Group 1 cells, as determined by assessing the number of cells in clusters 2, 4, and 8, compared to the total (**Supplementary Table S2**). IPA suggested that Group 1 monocytes exhibited heightened motility, neurotoxicity, and inflammatory functions that could contribute to the pathogenesis of neuroinflammatory diseases such as HIV-NCI.

We then examined DEGs specific for the molecular pathways identified by IPA. A heat map of some of these genes showed that Group 1 monocytes exhibited a similar gene expression signature with consistent upregulation of genes specific for the different molecular pathways, indicating that these clusters may share functional properties (**Figure 1C**). Conversely, Group 2 monocytes exhibited decreased expression of genes in those same pathways, indicating they may be more similar to each other and contribute less to neurotoxic and inflammatory processes. To characterize gene expression in monocytes within Group 1, we focused on DEGs that encoded cell surface proteins involved in migratory and inflammatory processes. ALCAM is a junctional adhesion protein that is critical to BBB transmigration of CD14^+^CD16^+^ monocytes (30, 34), and CD52, a GPI-anchored glycoprotein, is known to facilitate transendothelial migration in lymphocytes (35). Although the role of CD52 in monocytes remains incompletely characterized, it may contribute to adhesion (36). CD63 is a tetraspanin that is highly expressed on CD14^+^CD16^+^ monocytes and is associated with recruitment of monocytes to tissues during an inflammatory response (37). SDC2, a member of the syndecan family, regulates cell adhesion and migration (38), and has been linked to tumor cell invasiveness (39). The DEG data indicated that these genes were highly expressed in Group 1, with lower expression in Group 2 (**Figure 1C**). This was confirmed by violin plots showing relative expression of these DEGs (**Figure 1D**).

In addition to genes involved in migratory and inflammatory pathways, we also examined the expression of disease-associated microglia (DAM) genes. While some DAM are considered neuroprotective (40), many are implicated in the pathogenesis of several neurodegenerative diseases, including AD and amyotrophic lateral sclerosis (ALS). These are referred to as neurodegenerative microglia (MGnD) (41). A myeloid cell population similar to DAM has been identified in the cerebrospinal fluid (CSF) of PWH on antiretroviral therapy (ART), with these cells proposed to play a role in HIV-NCI (42). DAM markers such as *SPP1* and *APOE* (40, 41, 43) were among the most upregulated DEGs in monocytes from Group 1 (**Figure 1C**). Proteins encoded by these genes are reported to contribute to various CNS diseases (40, 41).

### ALCAM expression distinguishes a population enriched for Group 1 monocytes

**Figure 2:**
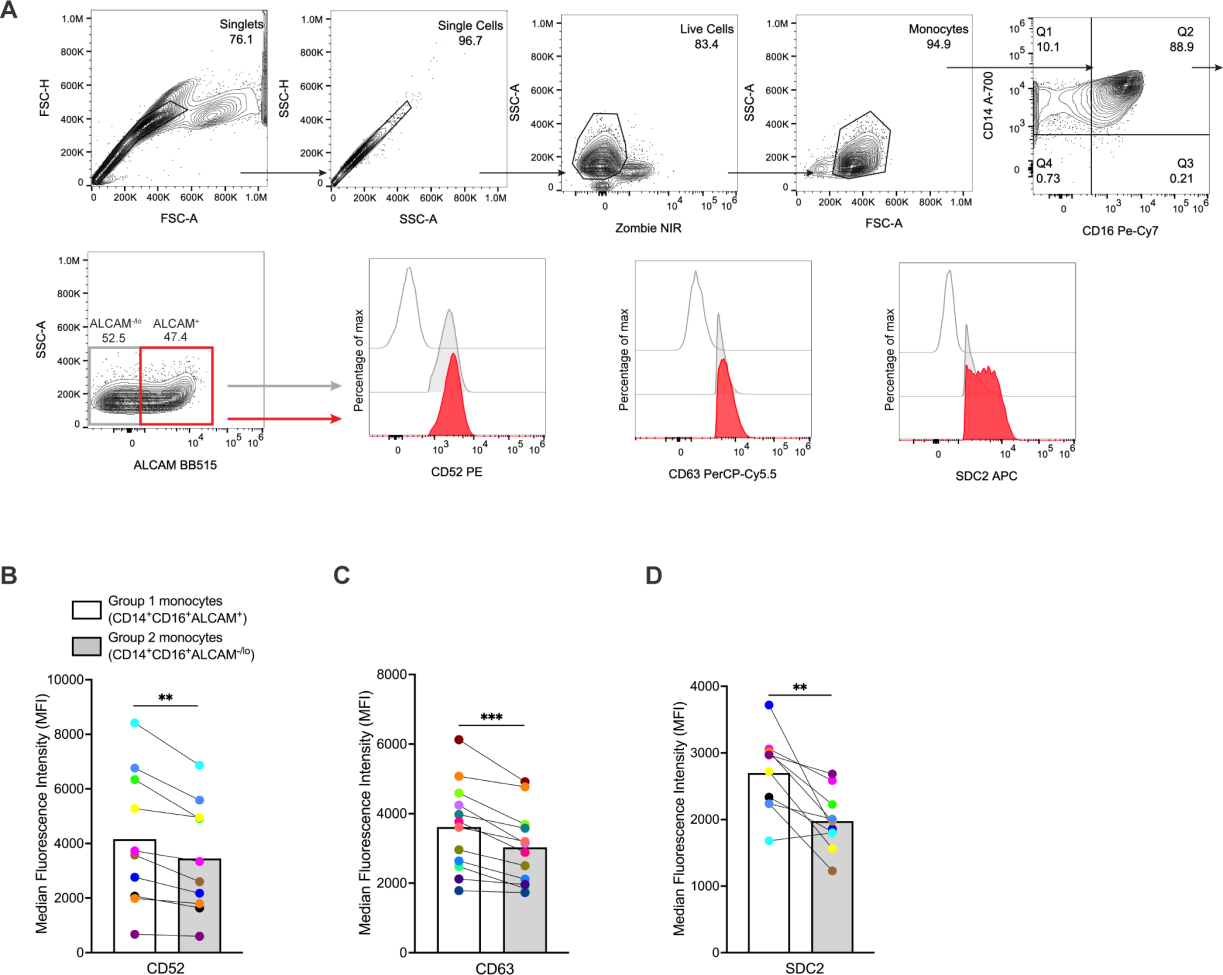
CD14^+^CD16^+^ monocytes from Group 1 clusters can be identified by ALCAM, CD52, CD63, and SDC2 surface expression as detected by flow cytometry. **(A)** Representative flow cytometry gating strategy for comparing MFIs of CD52, CD63, and SDC2 on CD14^+^CD16^+^ALCAM^+^ and CD14^+^CD16^+^ALCAM^-/lo^ monocytes. Red histograms represent CD14^+^CD16^+^ALCAM^+^ monocytes, gray histograms represent CD14^+^CD16^+^ALCAM^-/lo^ monocytes, and single line histograms represent FMO controls. **(B-D)** Composite MFI graphs of surface markers CD52, CD63, and SDC2 expressed on CD14^+^CD16^+^ALCAM^+^ (white bars) compared to CD14^+^CD16^+^ALCAM^-/lo^ (gray bars) monocytes. Each color and connecting line represent the MFIs for protein expression obtained from cells from each individual donor. Data are representative of n=10-12 independent donors. Significance was determined using paired t-tests (**p<0.01, ***p<0.001). Data are represented as mean ± SEM. MFI, median fluorescence intensity, FMO, fluorescence minus one.

To validate our scRNA-seq analysis and further characterize Group 1 monocytes, we examined, by flow cytometry, expression of cell surface proteins specific for genes enriched in Group 1 that play important roles in migration and inflammation. Freshly isolated CD14^+^ monocytes from ten independent donors were cultured non-adherently for three days to obtain mature CD14^+^CD16^+^ monocytes, as described previously (16, 17, 19, 30). Gene expression of several surface markers was higher in Group 1 monocyte clusters. Therefore, we stained for these in our flow cytometry experiments. CD14^+^CD16^+^ monocytes were labeled with fluorescent antibodies to ALCAM, CD52, CD63, and SDC2. ALCAM expression was used as an initial marker to identify a population enriched for Group 1 monocytes, with minimal or absent ALCAM expression identifying mainly Group 2 monocytes. These populations were distinguished by gating on ALCAM^+^ cells defined by FMO controls. We then used an additional gating strategy by examining ALCAM^+^ and ALCAM^-/lo^ cells for levels of CD52, CD63, and SDC2, which were expected to be elevated in Group 1 monocytes based on our scRNA-seq data (**Figure 2A**). MFIs of CD52, CD63, and SDC2 were compared between CD14^+^CD16^+^ALCAM^+^ and CD14^+^CD16^+^ALCAM^-/lo^ populations. Results showed that CD14^+^CD16^+^ALCAM^+^ cells have significantly higher MFIs for CD52 (p=0.0021), CD63 (p=0.0002), and SDC2 (p=0.003), than CD14^+^CD16^+^ALCAM^-/lo^ cells (**Figures 2B–D**), indicating that ALCAM^+^ cells have higher expression levels of these cell surface proteins and represent a monocyte population enriched for Group 1 monocytes.

### Cell sorted monocytes enriched for Group 1 cells exhibit increased expression of intracellular genes identified by scRNA-seq, including markers consistent with other neuroinflammatory cells

To validate and characterize further expression of various intracellular markers in Group 1 monocytes identified from scRNA-seq data, we performed FACS and subsequent qRT-PCR analyses of sorted cells enriched for Group 1 or Group 2 monocytes. We selected six DEGs that encode intracellular proteins associated with neurodegenerative and neuroinflammatory diseases, all of which were significantly increased in Group 1 compared to Group 2 monocytes. The genes examined were *CHI3L1*, and the DAM markers *APOC1, APOE, CTSB, LGALS3*, and *SPP1. APOC1* and *APOE* encode the apolipoproteins C1 and E, with *APOE* contributing significantly to AD development (44). *CHI3L1* encodes chitinase-3-like protein 1 and is upregulated in macrophages, microglia, and astrocytes associated with MS, AD, and HIV-associated dementia (45). *CTSB* encodes for cathepsin-B, a lysosomal protease involved in protein degradation and neurodegeneration (46, 47). *LGALS3* and *SPP1*, which encode galectin-3 and osteopontin proteins, respectively, have various signaling properties and can serve as chemoattractants for monocytes (48, 49). Collectively, *APOE, CTSB, LGALS3*, and *SPP1* are hallmark genes of DAM and MGnD (40, 41). We also analyzed *CLEC7A*, a C-type lectin and DAM marker, *FCGR2B*, which encodes the Fc fragment of IgG receptor IIb, and *DUSP1*, a dual-specificity protein phosphatase, as controls for detecting genes that were increased in Group 2 compared to Group 1 monocytes. We also quantified *TPT1*, a regulator of cell growth with homeostatic function often used to normalize qRT-PCR gene expression in monocytes (50, 51) as a control expected to be the same in both Group 1 and 2 monocytes.

**Figure 3:**
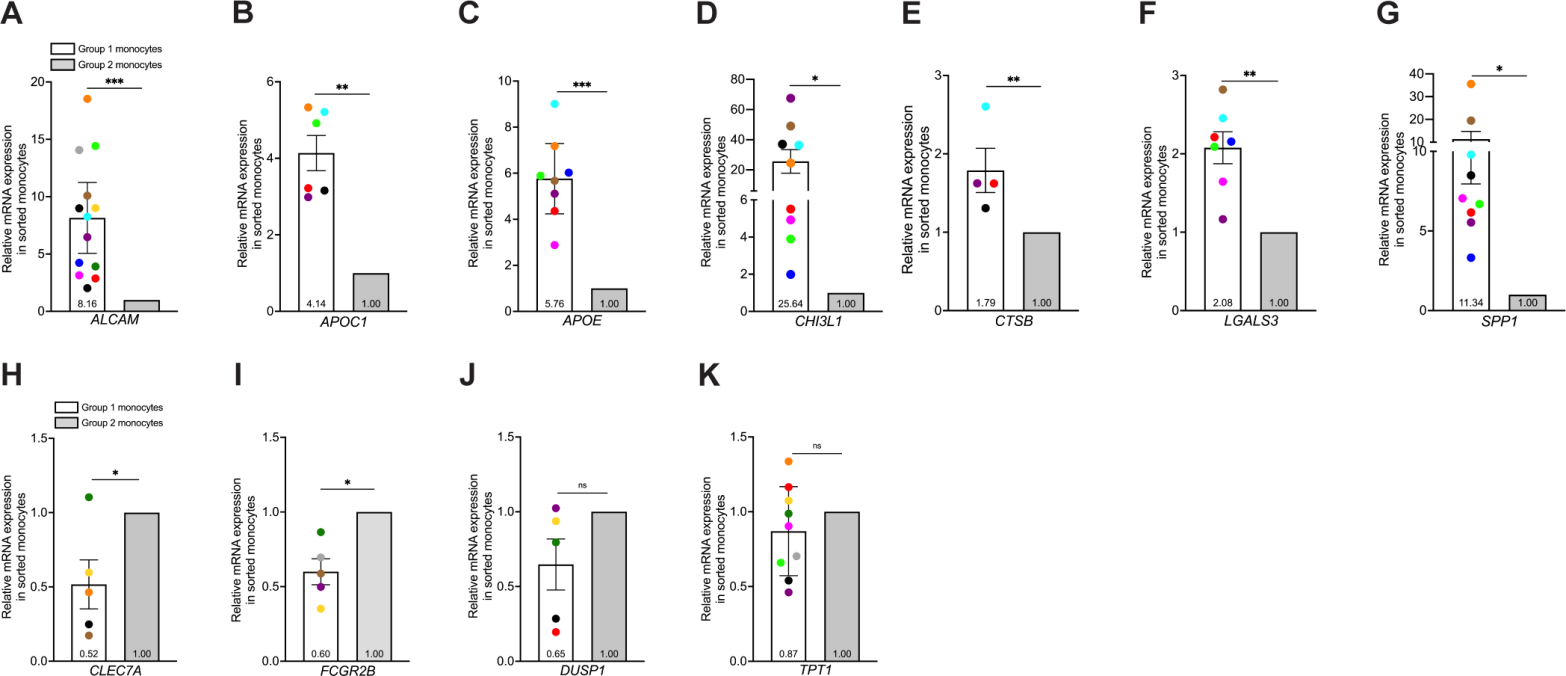
Isolation of Group 1 and Group 2 monocytes by cell sorting is validated by confirming DEGs identified by scRNA-seq that are increased in Group 1 or 2 monocytes by qRT-PCR. Group 1 and Group 2 cells were sorted by gating on CD14^+^CD16^+^ALCAM^+^ and CD14^+^CD16^+^ALCAM^-/lo^ monocytes. **(A-K)** Relative gene expression in cell sorted Group 1 (white bars) and Group 2 (gray bars) monocytes compared using the 2^-ΔΔCt^ method, normalized to the internal control gene *18S.* **(A-G)** Composite graphs of *ALCAM* (n=13), *APOC1* (n=6), *APOE* (n=8), *CHI3L1* (n=9), *CTSB* (n=4), *LGALS3* (n=7), and *SPP1* (n=9) that are increased in Group 1 monocytes. **(H-K)** Composite graphs of genes *CLEC7A* (n=5), *FCGR2B* (n=5), and *DUSP1* (n=5) analyzed as controls that were higher in Group 2 monocytes, and *TPT1* (n=9, p=0.227) as a control that was unchanged in both Group 1 (white bars) and Group 2 (gray bars) monocytes. Each color represents an individual donor. Each gene was tested in triplicate for each group. Significance was determined using one-sample t-tests (*p<0.05, **p<0.01, ***p<0.001, ns, not significant). Data are represented as mean ± SEM. DEGs, differentially expressed genes; FACS, fluorescence-activated cell sorting.

Monocytes from 13 independent donors were matured in culture as previously described, and stained for CD14, CD16, and ALCAM. Cells were sorted based on ALCAM positivity, and CD14^+^CD16^+^ALCAM^+^ and CD14^+^CD16^+^ALCAM^-/lo^ populations collected for RNA isolation. Analyses by qRT-PCR demonstrated that Group 1 monocytes (ALCAM^+^) exhibited higher expression of *APOC1* (p=0.001), *APOE* (p=0.0002), *CHI3L1* (p=0.014), *CTSB* (p=0.008), *LGALS3* (p=0.0018), and *SPP1* (p=0.016) compared to Group 2 monocytes (ALCAM^-/lo^) (**Figures 3B–G**). *CHI3L1* and *SPP1* showed large differences in expression between Group 1 and Group 2 monocytes, with fold changes at 25.64 and 11.34, respectively (**Figures 3D, G**). In addition, higher expression of *ALCAM* in Group 1 monocytes confirmed the effectiveness of FACS of CD14^+^CD16^+^ monocytes (**Figure 3A**). *CLEC7A* (p=0.043) and *FCGR2B* (p=0.01) were higher in Group 2 monocytes (**Figures 3H, I**). *DUSP1* (p=0.1078) was not significant but showed a trend for increased expression in Group 2 monocytes (**Figure 3J**). *TPT1* showed no significant difference (p=0.227) in expression between Group 1 and Group 2 monocytes (**Figure 3K**). These results confirm the use of ALCAM positivity alone to identify populations enriched in Group 1 or Group 2 monocytes that can be used to validate further the DEGs identified by our scRNA-seq data and characterize the gene expression profiles of Group 1 and Group 2 monocytes.

### Enriched Group 1 monocyte populations produce more inflammatory mediators

**Figure 4:**
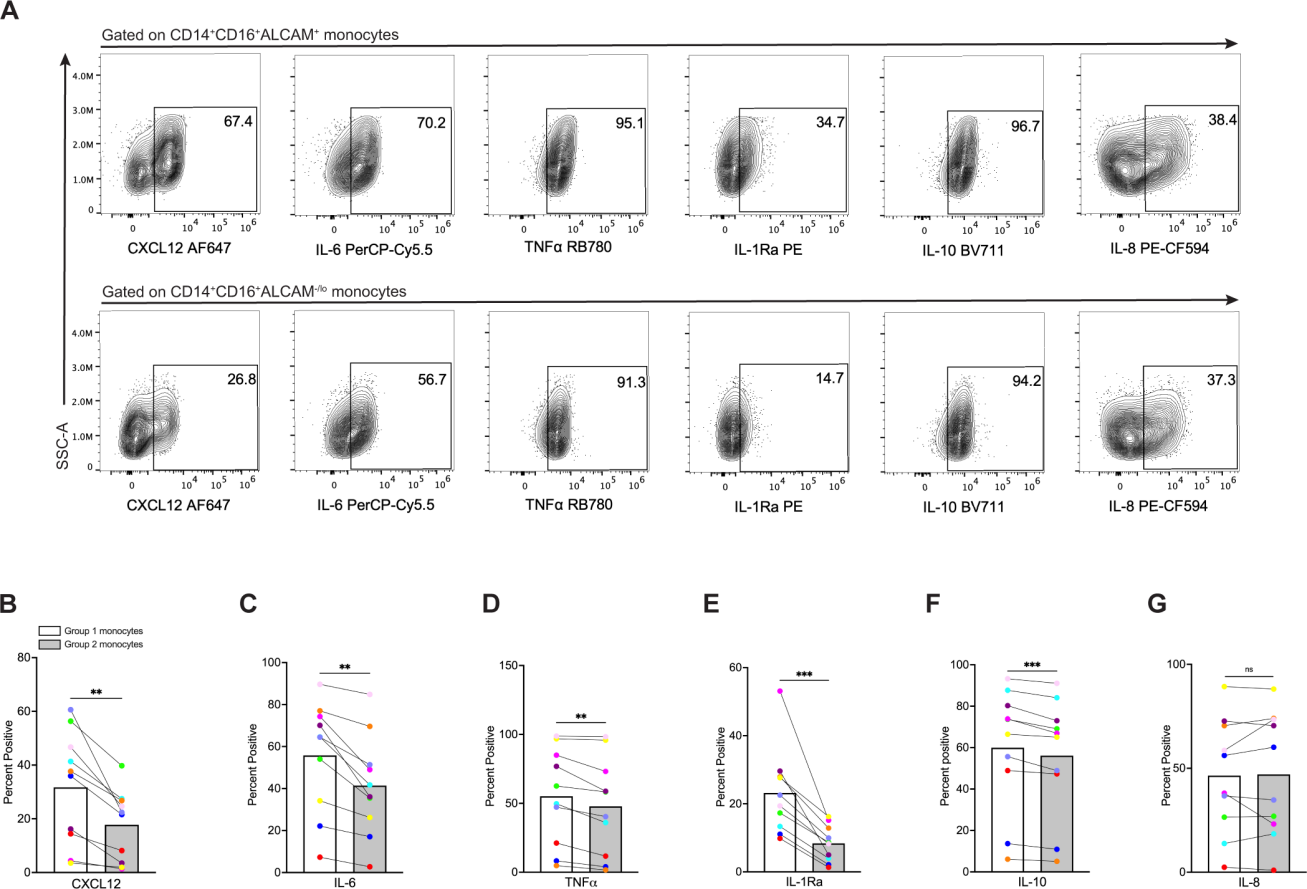
Group 1 monocytes produce more CXCL12, IL-6, TNFα, IL-1Ra, and IL-10 than Group 2 monocytes. **(A)** Representative flow cytometry plots of inflammatory mediators in CD14^+^CD16^+^ALCAM^+^ (Group 1) (top) and CD14^+^CD16^+^ALCAM^-/lo^ (Group 2) (bottom) monocytes. **(B-G)** Compiled graphs of the percent positive expression of CXCL12, IL-6, TNFα, IL-1Ra, IL-10, and IL-8 by Group 1 (white bar) and Group 2 (gray bar) monocytes. Each color and connecting line represent an individual donor. Data in **(B-G)** are representative of n=10 independent donors. Significance was determined using paired t-tests (**p<0.01, ***p<0.0001, ns, not significant). Data are represented as mean ± SEM.

We evaluated functional differences between Group 1 and Group 2 monocytes. CD14^+^CD16^+^ monocytes produce mediators that can either promote or suppress inflammation (52–55). To characterize inflammatory mediator production in Group 1 and Group 2 monocytes, we performed intracellular staining and flow cytometry. CD14^+^CD16^+^ monocytes were exposed to LPS for 24 hours, and then stained with antibodies to surface markers CD14, CD16, ALCAM, and intracellular CXCL12, IL-6, IL-8, and TNFα, chemokines and cytokines that contribute to inflammatory diseases, including HIV neuropathogenesis (56–59). We also evaluated IL-1Ra and IL-10, as these mediators possess anti-inflammatory properties, or are produced to downregulate and limit an ongoing inflammatory response (60, 61). Group 1 and Group 2 monocytes were defined as only CD14^+^CD16^+^ALCAM^+^ and CD14^+^CD16^+^ALCAM^-/lo^ for these experiments without including expression of the different surface proteins examined previously, due to the antibody panel complexity needed to analyze all the intracellular mediators in each cell, and the number of cells needed for proper controls. Percent expression of each mediator was then compared between CD14^+^CD16^+^ALCAM^+^ and CD14^+^CD16^+^ALCAM^-/lo^ populations (**Figure 4A**). Our results for ten independent donors demonstrated that Group 1 monocytes produced significantly more CXCL12 (p=0.002), IL-6 (p=0.002), TNFα (p=0.0103), IL-1Ra (p=0.0008), and IL-10 (p=0.0008), compared to Group 2 monocytes (**Figures 4B–F**). Interleukin-8 was similar between Groups (p=0.814, **Figure 4G**). Group 1 monocytes produced more IL-1Ra and IL-10 than Group 2 monocytes, contrary to our initial hypothesis that Group 2 monocytes may produce more of these anti-inflammatory mediators (**Figures 4E, F**).

### Group 1 monocytes express increased ROS compared to Group 2 monocytes

**Figure 5:**
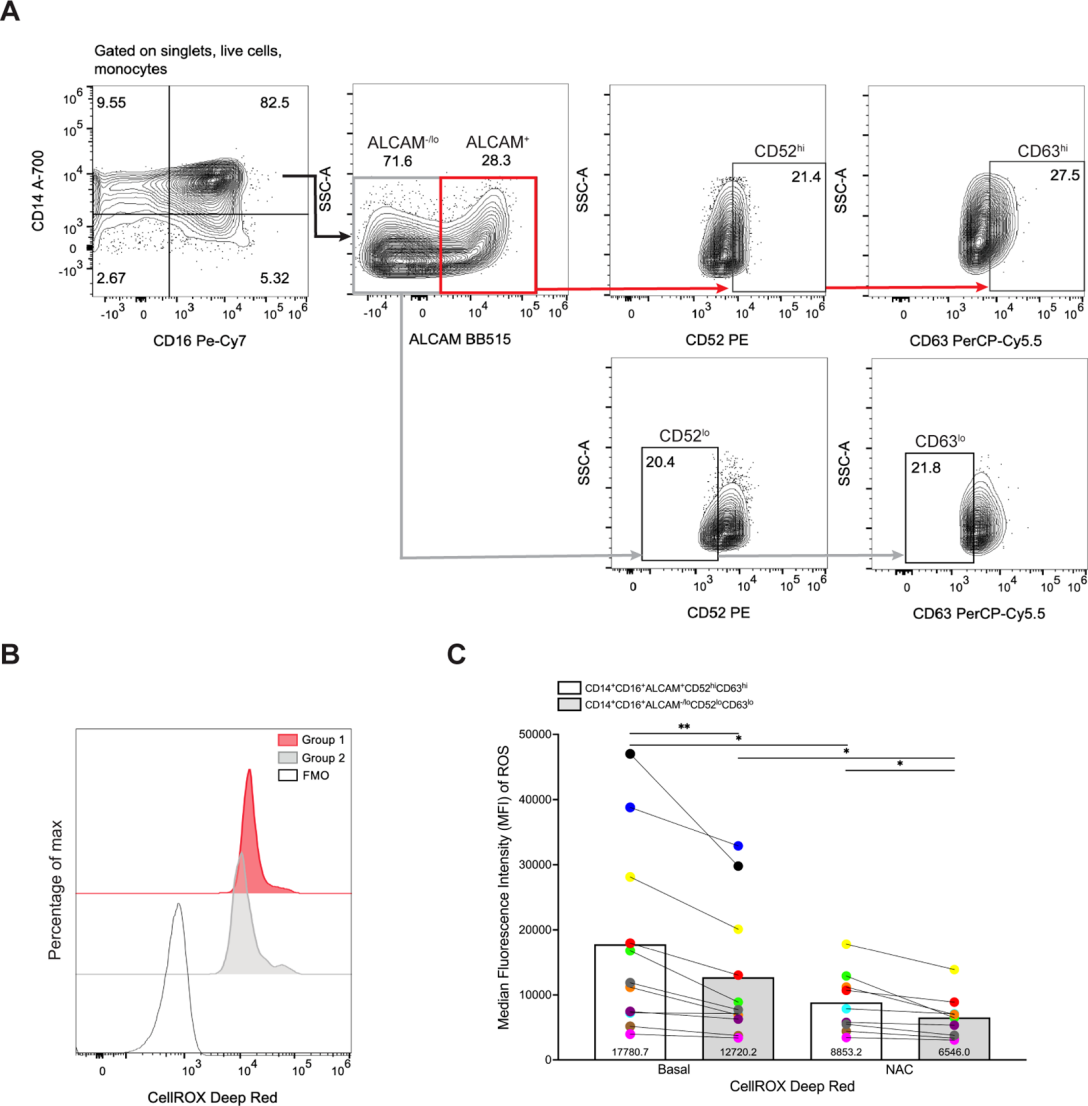
Group 1 monocytes produce more ROS than Group 2 monocytes. **(A)** Representative gating strategy for identifying Group 1 (CD14^+^CD16^+^ALCAM^+^CD52^hi^CD63^hi^) and Group 2 (CD14^+^CD16^+^ALCAM^-/lo^CD52^lo^CD63^lo^) monocytes. **(B)** Representative histogram overlays of ROS production by Group 1 and Group 2 monocytes as detected by CellROX MFI. The red histogram represents cells gated as Group 1 monocytes, the filled gray histogram represents cells gated as Group 2 monocytes, and the single line histogram represents the CellROX FMO control. **(C)** Compiled CellROX MFIs between Group 1 (white bar) and Group 2 (gray bar) monocytes at baseline (n=11), and with antioxidant NAC (n=9). Experiments were performed with 3-5 replicates per condition and controls for each donor. Each color and connecting line represent an individual donor. Significance was determined using paired t-tests (*p<0.05, **p<0.01). Data are represented as mean ± SEM. ROS, reactive oxygen species, MFI, median fluorescence intensity, FMO, fluorescence minus one, NAC, N-Acetylcysteine.

CD14^+^CD16^+^ monocytes generate ROS (62) that can contribute to neuropathogenic diseases, such as HIV-NCI (63–65). Our scRNA-seq analysis demonstrated that Group 1 monocytes have decreased levels of superoxide dismutase 2 (SOD2) (**Figure 1C**), an important antioxidant that neutralizes ROS (66). Thus, we examined whether Group 1 monocytes from eleven independent donors produce more ROS than Group 2 monocytes using flow cytometry. CD14^+^CD16^+^ monocytes were incubated with CellROX, a dye that detects cytoplasmic ROS. As controls, cells were incubated with TBHP, a compound that induces ROS, and NAC, an antioxidant that neutralizes ROS, prior to addition of CellROX. To delineate Group 1 from Group 2 monocytes further, we performed sequential gating where ALCAM^+^ and ALCAM^-/lo^ cells were gated for high and low expression of CD52 and CD63, respectively (**Figure 5A**). Using this gating strategy, Group 1 monocytes were classified as CD14^+^CD16^+^ALCAM^+^CD52^hi^CD63^hi^, and Group 2 monocytes as CD14^+^CD16^+^ALCAM^-/lo^CD52^lo^CD63^lo^. We then compared basal ROS between the two groups of cells by MFIs (**Figure 5B**). Our results showed that Group 1 monocytes produce significantly more ROS at baseline compared to Group 2 monocytes (p=0.007, **Figure 5C**). NAC effectively inhibited ROS production in both Group 1 (p=0.031) and Group 2 monocytes (p=0.027, **Figure 5C**).

### Group 1 monocytes preferentially transmigrate in greater numbers across the BBB to CCL2 when compared to Group 2 monocytes

**Figure 6:**
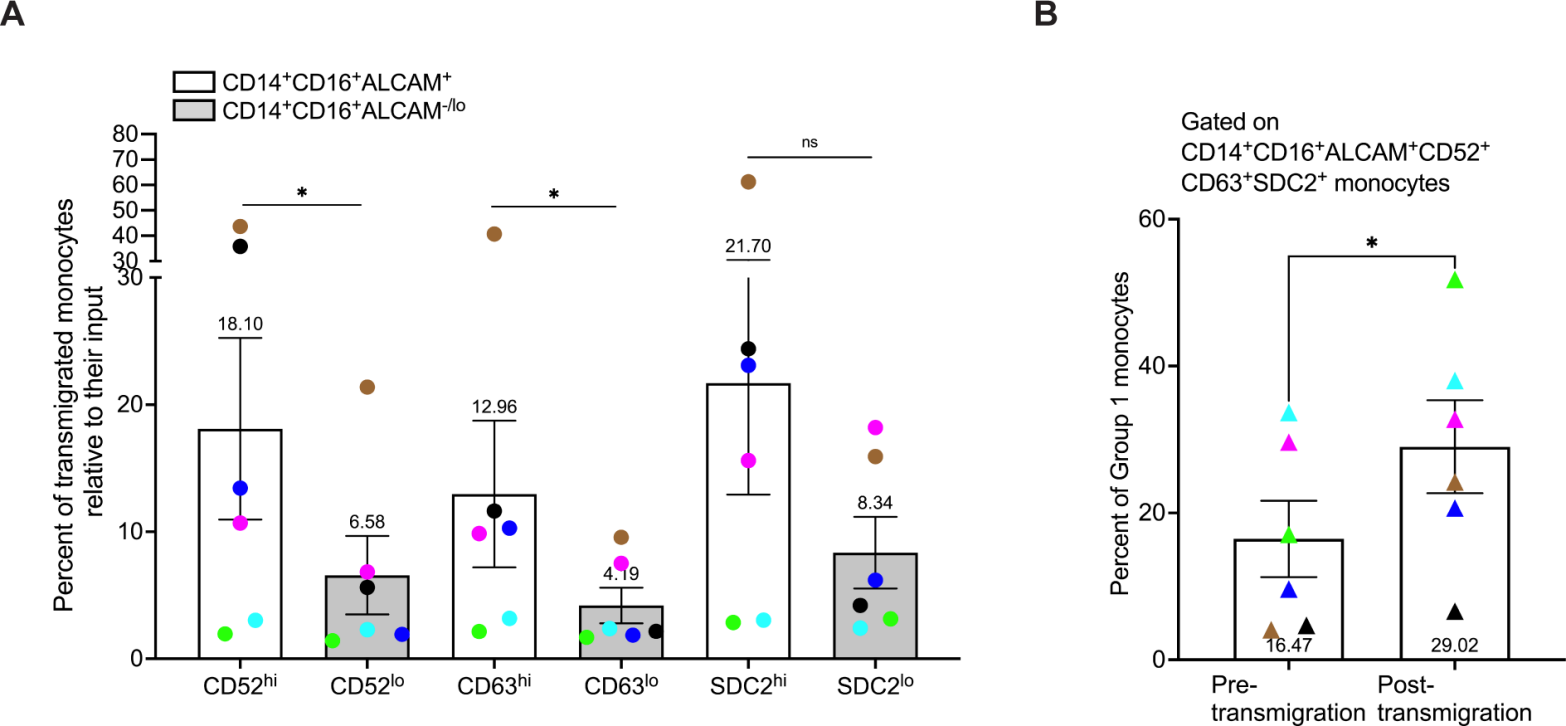
Group 1 monocytes preferentially transmigrate across the BBB in greater numbers than Group 2 monocytes. CD14^+^CD16^+^ monocytes were cultured for 3 days non-adherently *in vitro*, added to our human *in vitro* BBB co-culture model, and allowed to transmigrate for 24 hours to CCL2. Group 1 monocytes were defined by gating on CD14^+^CD16^+^ALCAM^+^ monocytes that had high expression (top 20^th^ percentile) of CD52, CD63, and SDC2. Group 2 was defined by gating on CD14^+^CD16^+^ALCAM^-/lo^ cells that had low expression (bottom 20^th^ percentile) for the same proteins. Each marker was evaluated separately on CD14^+^CD16^+^ALCAM^+^ and CD14^+^CD16^+^ALCAM^-/lo^ cells. **(A)** Composite graph of the number of each Group of monocytes post-transmigration divided by the number of each Group of monocytes in the pre-transmigration input population, expressed as the percent of Group 1 (white bar) or Group 2 (gray bar) monocytes, relative to their input that transmigrated across the BBB. **(B)** Percent of Group 1 monocytes in the pre-transmigration input population (left) and the post-transmigration population (right) using sequential gating for expression of CD52, CD63, and SDC2 on CD14^+^CD16^+^ALCAM^+^ cells. Experiments were performed with n=6 independent donors, with quadruplicate co-culture replicates for samples stained with the full panel of antibodies and each FMO control. Each color represents an individual donor. Significance was determined using a Wilcoxon matched-pairs signed rank test (*p<0.05, ns, not significant). Data are represented as mean ± SEM.

CD14^+^CD16^+^ monocytes have been shown to preferentially transmigrate across the BBB compared to classical and non-classical monocytes (16–19, 30). Our findings in this study indicate that Group 1 monocytes are a subpopulation of CD14^+^CD16^+^ monocytes that produce more ROS and inflammatory mediators, suggesting that entry of these cells into the CNS would contribute significantly to HIV-NCI and other neuroinflammatory diseases. Thus, we examined whether Group 1 monocytes preferentially transmigrated across the BBB compared to Group 2 monocytes using a human *in vitro* co-culture BBB model that has been used extensively to characterize mechanisms of monocyte transmigration (16, 19, 30, 33, 34, 67). Group 1 monocytes were defined as CD14^+^CD16^+^ALCAM^+^CD52^hi^CD63^hi^SDC2^hi^, and Group 2 monocytes as CD14^+^CD16^+^ALCAM^-/lo^CD52^lo^CD63^lo^SDC2^lo^. Pre- and post-transmigrated CD14^+^CD16^+^ monocytes were first gated for ALCAM^+^ or ALCAM^-/lo^ cells, and then sequential gating was performed to assess high or low expression of each individual additional marker on ALCAM^+^ and ALCAM^-/lo^ cells, respectively. Since the number of cells post-transmigration is limited, we did not have a sufficient number of cells to perform sequential gating to analyze high expression of all Group 1 markers on transmigrated ALCAM^+^ cells. Thus, in a separate analysis, after gating for ALCAM^+^ monocytes, we performed sequential gating for overall positive expression of CD52, CD63, and SDC2. Our results using gating for each marker individually showed that Group 1 monocytes defined by ALCAM positivity and high expression of CD52 (p=0.0312) or CD63 (p=0.0312) preferentially transmigrated across the BBB compared to Group 2 monocytes, as expressed by the percent of transmigrated Group 1 cells relative to their input (**Figure 6A**). While there was no significance in transmigration of cells with high compared to low expression of SDC2 (p=0.219), there was a trend toward increased transmigration of Group 1 cells with high expression of this marker. This preferential transmigration of Group 1 monocytes was confirmed by sequential gating for expression of all markers on ALCAM^+^ cells pre-transmigration compared to post-transmigration (p=0.031, **Figure 6B**). These findings suggest that Group 1 monocytes have a higher propensity to transmigrate across the BBB into the CNS than Group 2 monocytes, indicating that they are primed to enter the CNS and be major contributors to neuropathogenesis.

## Discussion

Despite advances in characterization of CD14^+^CD16^+^ monocytes and their roles in disease pathogenesis, these cells remain significantly understudied. This subset is implicated in various inflammatory conditions, including HIV-NCI (8–11), as well as other neuroinflammatory and cardiovascular diseases (20, 22, 23), highlighting the need for a comprehensive characterization of these cells. After scRNA-seq analysis of human CD14^+^CD16^+^ monocytes matured *in vitro*, we identified nine distinct clusters in this monocyte subset. Through DEG and IPA analyses, we determined that these clusters separated into two groups, with one group having a more migratory and inflammatory phenotype. We propose that these cells, termed Group 1 monocytes, have the potential to contribute to neuroinflammation, specifically HIV-NCI, and can be targeted as a therapy for this and other CNS pathologies.

Human peripheral blood monocytes exhibit considerable heterogeneity (68–70). With the significant advancement of single-cell analysis, several studies have reported more than three subsets of total monocytes (26–28). Many studies primarily characterized total monocyte subsets from PBMCs based on CD14 and CD16 expression alone, often combining intermediate and non-classical monocytes (29, 71). In addition, studies have not performed extensive analysis of the intermediate monocyte subset. For example, one study used scRNA-seq on cell-sorted monocytes from PBMCs of one donor, identifying four monocyte subsets, including classical and non-classical subsets and two novel ones (26). However, intermediate monocytes were not defined as a distinct subset but rather dispersed across all four subsets. Thus, the heterogeneity within the CD14^+^CD16^+^ monocyte subset was not addressed. Few studies have specifically focused on the phenotypic and functional characterization of CD14^+^CD16^+^ monocytes. In one study, researchers used scRNA-seq to identify three subpopulations within the CD14^+^CD16^+^ monocyte subset, two of which were associated with an increased risk of CAD in females (72). This study also found higher expression of *CD52* and *LGALS3* in some of the clusters, suggesting heterogeneity in CD14^+^CD16^+^ monocyte subpopulations, consistent with our findings. Another scRNA-seq study of cell-sorted CD14^+^CD16^+^ monocytes from PBMCs of individuals with active tuberculosis defined six subpopulations within this subset, highlighting its complexity and diversity (27). A potential limitation of these studies is that they were conducted using PBMCs, of which CD14^+^CD16^+^ monocytes represent a small fraction. Therefore, obtaining enough CD14^+^CD16^+^ monocytes from PBMCs is challenging due to the minimal numbers of these cells in the peripheral circulation. Optimizing sequencing depth, especially for cell groups with distinct subpopulations, is highly dependent on cell number in scRNA-seq analysis (73). Our scRNA-seq analysis, facilitated by unique isolation and culturing methods, enabled the examination of a large number of CD14^+^CD16^+^ monocytes, thus facilitating detection of greater heterogeneity and subpopulations within this monocyte subset. In our study, we delineated nine distinct clusters in the CD14^+^CD16^+^ monocyte subset. This is the first study, to our knowledge, to characterize these subpopulations in detail and identify a specific group of CD14^+^CD16^+^ monocytes with phenotypic and functional properties that could enhance the ability of these cells to mediate neuroinflammation and CNS damage. As scRNA-seq analysis becomes increasingly feasible and accessible, further identification and characterization of subpopulations within the CD14^+^CD16^+^ monocyte subset are anticipated.

Our scRNA-seq data characterized two distinct groups of cells within the CD14^+^CD16^+^ monocyte subset, with one group having a more migratory and inflammatory phenotype (Group 1) compared to the other (Group 2). Unlike conventional scRNA-seq analysis that rely on canonical markers to categorize cell types from PBMCs, our approach was somewhat novel. In our study, we only analyzed one type of cell, CD14^+^CD16^+^ monocytes. Clusters identified by scRNA-seq did not each express canonical markers that characterize different cell types. Rather, the genes expressed in all clusters were similar, with the level of expression varying among different clusters. Thus, DEGs were identified by comparing the expression of a gene in a specific cluster to the expression of that gene in all other clusters combined. As a result, we were able to group clusters into two groups based on DEGs that were upregulated or downregulated to a similar extent in each cluster. IPA analysis of DEGs identified many increased migratory and inflammatory pathways in Group 1 compared to Group 2 cells. Thus, our subsequent analysis was focused on genes involved in these pathways. There was high differential expression of *ALCAM* in Group 1 compared to Group 2 monocytes. ALCAM is an adhesion protein increased on CD14^+^CD16^+^ monocytes, crucial for transmigration across the BBB (30, 74). Additionally, Group 1 monocytes exhibited elevated differential expression of *CD52, CD63*, and *SDC2*. While high *CD52* and *CD63* differential expression was specific for Group 1 clusters, *SDC2* was more broadly expressed in all clusters. ALCAM, CD52, CD63, and SDC2 surface proteins were then examined for their ability to be used as markers to distinguish Group 1 and Group 2 monocytes by flow cytometry. Monocyte populations enriched in Group 1 clusters were found to be ALCAM^+^, while those enriched in Group 2 clusters did not express, or had low levels, of ALCAM. In addition to ALCAM, more delineated populations of Group 1 monocytes also exhibited higher MFIs for CD52, CD63, and SDC2, compared to Group 2 cells. Overall, the increased expression of all of these surface proteins involved in migration and inflammation characterize Group 1 monocytes, suggesting the potential ability of these cells to cross the BBB preferentially and mediate neuropathogenesis. In conjunction with ALCAM positivity, these markers serve not only to identify and characterize Group 1 monocytes but also offer promising avenues for therapeutic targets.

We validated further the scRNA-seq data using FACS to isolate populations enriched in Group 1 or 2 monocytes followed by qRT-PCR gene expression analysis. Our findings indicated that Group 1 monocytes, sorted based on ALCAM positivity, showed upregulated gene expression for *APOC1*, *APOE*, *CHI3L1*, *CTSB*, *LGALS3*, and *SPP1* (**Figure 3**). *APOC1* and *APOE* play roles in regulating lipid metabolism in the CNS (75). Additionally, upregulated *APOE* is characteristic of DAM and MGnD in the CNS (40, 41), implicating innate immune involvement in various neurological disorders. A recent scRNA-seq study of CSF from PWH on ART, identified a myeloid cell subset that they termed “microglia-like” with a gene expression profile characteristic of neurodegenerative DAM, which the authors hypothesized contributes to increased neuronal injury in HIV neuropathogenesis (42). The upregulation of *APOE* and *TREM2* (triggering receptor expressed on myeloid cells 2) in our data supports the hypothesis that Group 1 monocytes can contribute to neuroinflammation once these cells enter the CNS and differentiate into macrophages that have a similar phenotype to DAM, MGnD, and HIV-associated microglia-like cells, that are characterized by APOE activation of TREM2. This will be the focus of future studies. *LGALS3* was also upregulated in Group 1 monocytes. Galectin-3, encoded by *LGALS3*, is produced by human monocytes and can act as a monocyte/macrophage chemoattractant (76), and is another signature marker for neurodegenerative DAM (77). *CTSB*, which is neurotoxic (78) and increased in HIV-infected monocyte-derived macrophages in HIV-NCI (78, 79), was also increased in Group 1 cells, suggesting a potential detrimental function mediated by overexpression of this protein. *CHI3L1* was significantly upregulated in Group 1 monocytes and its expression is upregulated in neurological diseases such as MS and AD (45, 80, 81). It is also neurotoxic (81, 82) and inhibits neurogenesis (83, 84), suggesting its role in neuroinflammation and neurodegeneration. Similarly, *SPP1* was also significantly upregulated in Group 1 and is involved in regulating cytokine secretion and in monocyte chemoattraction (85). Studies in an animal model of HIV neuropathogenesis have demonstrated the role of osteopontin (SPP1) in the recruitment and accumulation of monocytes in the CNS (85), while elevated expression of the osteopontin receptor on CD14^+^CD16^hi^ monocytes was also linked to encephalitis (86). Additionally, osteopontin is increased in the CSF and CNS of individuals with HIV-NCI (87). Increased *SPP1* expression in Group 1 monocytes may enhance the ability of these cells, once they enter the CNS, to recruit additional peripheral monocytes contributing to neuroinflammation and HIV-NCI. Together, the upregulated markers in Group 1 monocytes suggest a gene expression signature consistent with other neuroinflammatory and neurodegenerative-associated cells.

Initially, we hypothesized that Group 1 monocytes would exhibit higher levels of inflammatory cytokines, and that Group 2 monocytes might display a more anti-inflammatory profile. Additionally, it was possible that Group 1 monocytes would be more receptive to LPS. Consistent with existing characterizations of inflammatory monocytes, we found that Group 1 monocytes produced elevated levels of CXCL12, IL-6, and TNFα. However, unexpectedly, we also found high levels of IL-1Ra and IL-10 in Group 1 monocytes. Since IL-1Ra counteracts the effects of IL-1β, a classic inflammatory cytokine that was not analyzed in this study, it is plausible that elevated IL-1Ra levels observed in our study may result from increased IL-1β expression (61). We also showed increased IL-10 levels in Group 1 monocytes. CD14^+^CD16^+^ monocytes are the major producers of IL-10 (54). A potential explanation for this finding is that IL-10 often plays an autoregulatory role by downmodulating the production of inflammatory cytokines (60, 88, 89). A limitation of our study is that cytokine analyses were performed at a single time point, specifically 24 hours after LPS stimulation. Previous studies reported that IL-10 peaks 24-48 hours after LPS stimulation in monocytes, unlike IL-6, TNFα, and IL-8, which peak earlier (90, 91). Our findings may be attributed to the 24-hour incubation with LPS, which may have induced high amounts of IL-1Ra and IL-10 to mitigate the effects of inflammatory mediators produced early after LPS treatment. Future cytokine analyses will be tailored to the optimal stimulus and kinetics for each cytokine’s detection, enabling a more comprehensive understanding of their roles in Group 1 monocyte-mediated inflammation.

CD14^+^CD16^+^ monocytes serve as significant sources of ROS that can contribute to neuroinflammation and subsequent disease progression. Elevated ROS levels are a hallmark of HIV infection (92–94), and ROS in the CNS contributes to HIV-NCI (63, 65, 95) and other neurodegenerative diseases (96–98). However, the extent of ROS production by infiltrating CD14^+^CD16^+^ monocytes and their precise contributions to neuroinflammatory pathogenesis remain incompletely understood. Having identified subpopulations of CD14^+^CD16^+^ monocytes with increased expression of genes associated with heightened inflammation and pathogenicity, and decreased levels of *SOD2*, we examined whether ROS production by Group 1 monocytes could be contributing to neuropathogenic effects of these cells. Our analysis showed that Group 1 monocytes exhibited higher ROS production compared to Group 2 cells under steady-state conditions that could be mitigated by the antioxidant NAC. While our study did not examine ROS production in an inflammatory context, this high basal level expression could be further amplified by a neuroinflammatory environment upon the infiltration of these cells into the CNS. In PWH on ART, and in animal models of HIV infection in the presence of ART, CNS viral reservoirs are detected in macrophages, some of which are proposed to derive from peripheral blood monocytes that have transmigrated across the BBB (12, 99, 100). Group 1 monocytes with higher baseline ROS levels could differentiate into macrophages once they infiltrate the CNS, and ROS production by these cells could potentially contribute to the propagation and exacerbation of neuroinflammation, ultimately resulting in neuronal damage.

Group 1 monocytes exhibited preferential transmigration across a human *in vitro* BBB model compared to Group 2 monocytes. In our transmigration experiments, we assessed Group 1 monocytes both by high expression of individual surface markers on CD14^+^CD16^+^ALCAM^+^ cells, and by sequential gating to assess the overall, rather than high, expression of all markers on CD14^+^CD16^+^ALCAM^+^ cells. We found Group 1 monocytes to be enriched post-transmigration using both methods of analysis. Our previous studies emphasized the important role of ALCAM in facilitating the transmigration of both uninfected and HIV infected CD14^+^CD16^+^ monocytes across this BBB model (30, 34, 74). Our current data demonstrate that Group 1 monocytes, characterized by ALCAM positivity and enhanced expression of other surface markers, exhibit a higher propensity to transmigrate across the BBB compared to Group 2 cells. These findings strongly suggest that Group 1 monocytes, compared to Group 2 cells, are more likely to infiltrate the CNS. Additionally, upon re-analyzing scRNA-seq data from our previous study on HIV infected CD14^+^CD16^+^ monocytes (31), we also found nine clusters in infected cells that could be divided into two groups with gene expression profiles resembling those of uninfected cells examined in this study (data not shown). This suggests that in PWH, Group 1 monocyte entry into the CNS will be a major contributor to HIV-NCI, warranting further examination in future studies. Overall, our characterization of Group 1 monocytes suggests a potential avenue for selectively targeting these cells to mitigate HIV-NCI, as well as other neuroinflammatory diseases. Subsequent studies will characterize CD14^+^CD16^+^ monocyte subpopulations in the context of HIV, including the identification of Group 1 and Group 2 CD14^+^CD16^+^ monocytes in PBMCs from PWH.

Our study has certain limitations. We did not optimize inflammatory mediator experiments based upon their kinetics or the ideal stimuli for each cytokine. Additionally, intracellularly detected mediators may not always correspond accurately to secreted factors. Thus, future studies will incorporate more detailed kinetics and stimuli for inflammatory mediators, as well as assess the secretion of these mediators using ELISA assays. While a strength of our study is that CD14^+^ monocytes were isolated and matured *in vitro* to obtain large numbers of CD14^+^CD16^+^ monocytes, this is also a limitation. We did not use PBMCs due to the limited amounts of CD14^+^CD16^+^ monocytes in the peripheral circulation. Manipulating and culturing cells can potentially alter their properties by modifying their microenvironment or inducing biochemical changes that may affect the expression of surface markers or cytokines (101). However, cell manipulation is an inherent aspect of any culture method, whether involving primary cells or established cell lines. Our *in vitro* culturing system has been rigorously tested and validated, consistently producing matured cells that express all traditional markers of CD14^+^CD16^+^ monocytes (17, 18). Future studies will be important to identify and characterize these two monocyte groups in PBMCs, likely requiring leukapheresed blood to obtain sufficient numbers of cells. In addition, studies will be performed to identify these groups in CD14^+^CD16^+^ monocytes infected *in vitro* with HIV, as well as in PBMCs of PWH. Despite these limitations, our findings provide valuable understanding of the heterogeneity of CD14^+^CD16^+^ monocytes and the potential role of subpopulations within this monocyte subset in neuroinflammatory diseases. This will facilitate development of targeted therapeutic interventions aimed at mitigating their pathogenic effects within the CNS.

## Data Availability

The original contributions presented in the study are included in the article/**Supplementary Material**, further inquiries can be directed to the corresponding author.
