# Supplementary figures and images for: Single-cell analysis of CD14+CD16+ monocytes identifies a subpopulation with an enhanced migratory and inflammatory phenotype
Source: Front Immunol. 2025 Feb 20;16:1475480. doi: 10.3389/fimmu.2025.1475480 (PMC11883828; doi:10.3389/fimmu.2025.1475480)

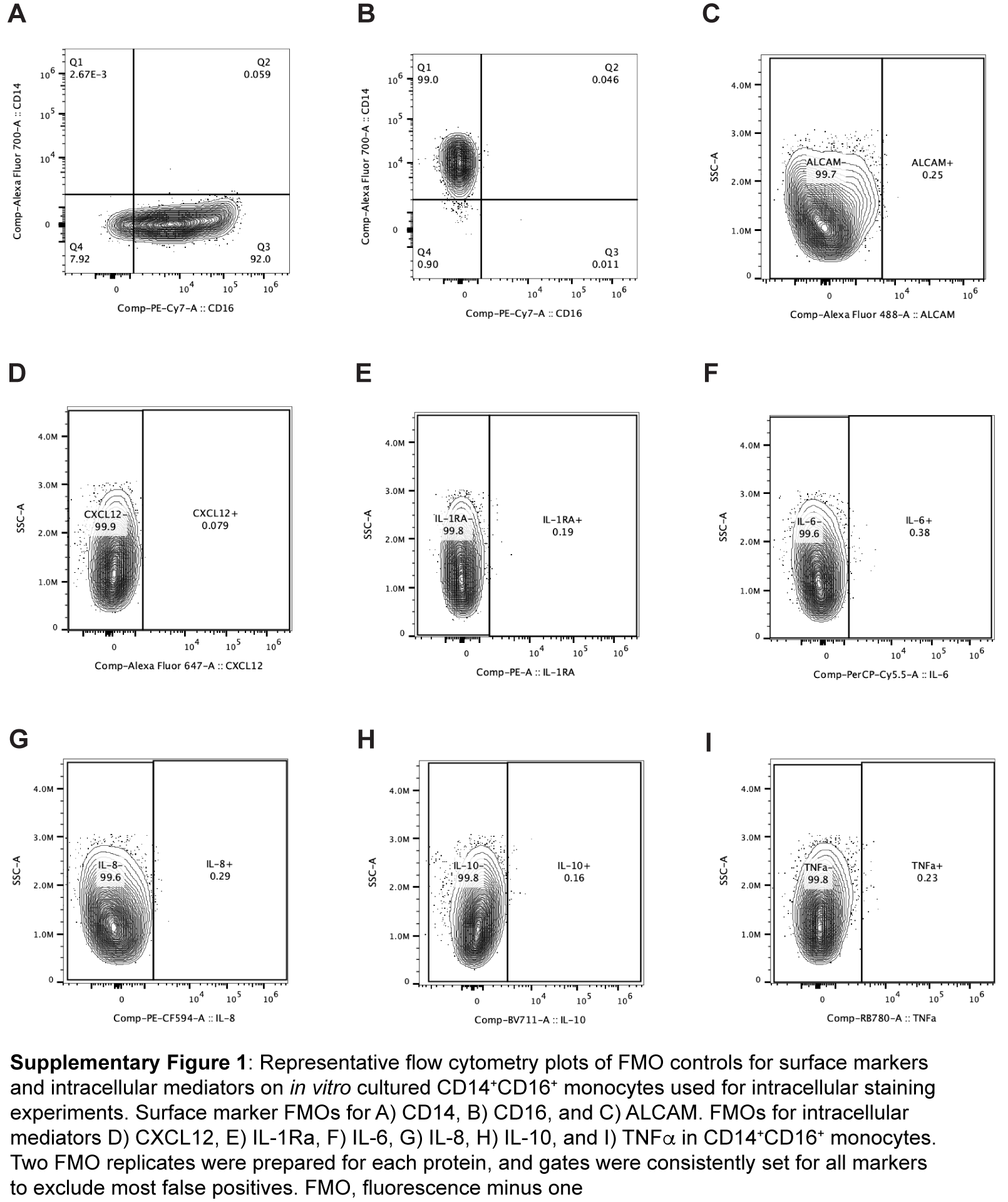

Supplement: Supplementary file 3 [file Image1.tif]
